# The experience of a tertiary referral center with laparoscopic pyelolithotomy for large renal stones during 18 years

**DOI:** 10.1038/s41598-023-50331-w

**Published:** 2023-12-28

**Authors:** Amir Hossein Kashi, Nasser Simforoosh, Akbar Nouralizadeh, Seyed Mohammad Ghasemi, Amirhossein Nayebzade, Milad Bonakdar Hashemi, Reza Valipour, Abbas Basiri, Ali Tabibi, Homayoun Zargar, Mehdi Dadpour, Hamidreza Rouientan, Behzad Narouie

**Affiliations:** 1 Urology and Nephrology Research Center (UNRC), Department of Urology, Shahid Labbafinejad Medical Center, Shahid Beheshti University of Medical Sciences (SBMU), Tehran, Iran; 2 Department of Urology, Tehran Medical Sciences Branch, Islamic Azad University, Tehran, Iran; 3 Department of Urology, Western Health, Melbourne, Australia; 4 Department of Urology, Royal Melbourne Hospital, University of Melbourne, Melbourne, Australia; 5 Department of Urology, Zahedan University of Medical Sciences, Zahedan, Iran

**Keywords:** Urology, Medical research

## Abstract

This study aimed to evaluate the outcomes of laparoscopic pyelolithotomy, including its efficacy and feasibility in treatment of large renal stones. All patients who underwent laparoscopic pyelolithotomy operations in a referral center were enrolled from 2003 to 2020. The final analysis included 436 patients. The total stone free rate was 88.3% and the stone-free rate for staghorn/multiple stones versus other types of stones was 81% vs. 91% (*P* = 0.002). Likewise, the total operation duration was 158 ± 50 and the operation duration for staghorn/multiple stones versus other types of stones was 171 ± 51 min vs. 153 ± 49 min (*P* < 0.001). The operation duration (169 ± 51 vs. 155 ± 58 vs. 155 ± 42 min) and hospitalization (4.5 ± 2.3 vs. 4.0 ± 2.2 vs. 3.6 ± 1.8) decreased with increasing the surgeons' experience over time. The outcomes of laparoscopic pyelolithotomy for children versus adults versus geriatric patients and in patients with normal versus abnormal kidney anatomy did not reveal statistically significant differences. Laparoscopic pyelolithotomy could be employed as an alternative surgical approach for patients with large kidney stones of any age or with kidney abnormalities provided that appropriate expertise is available to carry out the procedure.

## Introduction

Urolithiasis affects about 10% to 20% of the population worldwide and has increasing prevalence and recurrence rates in both developed and developing countries^1^. Kidney stones disease can cause morbidities, including cardiovascular diseases, chronic kidney disease, or renal failure^2–4^. Although open surgery has been noted as a historically primary treatment modality for kidney stone disease, its indications have significantly decreased owing to the introduction of minimally invasive interventions, including extracorporeal shockwave lithotripsy (ESWL), percutaneous nephrolithotomy (PCNL), laparoscopic pyelolithotomy (LPL), and ureteroscopy (URS), as well as the involvement of the medical approaches^5–8^.

Despite different minimally invasive options for kidney stone removal such as ESWL, PCNL, LPL, and URS^5,6^ the management of complex and large stones is a challenge for many urologists. PCNL has long been used as a primary treatment for large and complex renal calculi because of its high stone-free rate. However, bleeding, injury to collecting systems or contagious organs, sepsis, and renal loss have been reported^9–11^. LPL is another minimally invasive modality that has recently been adopted by expert centers, following its first report in 1994 by Gaur et al.^12^. According to the latest European Association of Urology guidelines, LPL is considered an appropriate alternative to PCNL and is preferred in cases where ESWL and PCNL are not feasible^1^. Irrespective of several studies on LPL, there are still questions regarding the indications and efficacy of LPL^7,13^. This procedure is advised to be performed by skilled surgeons to achieve excellent outcomes in terms of stone free status, operative time, postoperative kidney function, hospitalization duration, and complications^14,15^.

In this study, we aimed to evaluate the most suitable candidates for this procedure and the outcomes of LPL operations for the treatment of renal stones during 18 years at a tertiary referral center.

## Materials and methods

This was a longitudinal study of prospectively collected data from all patients who underwent LPL between 2003 and 2020 in the Shahid Labbafinejad Hospital, Tehran, Iran. The Labbafinejad Hospital is a tertiary referral center in Iran enrolling more than 1000 PCNL operations a year. Therefore, the included patients included only a small fraction of patients who were candidates for kidney stone operations. The inclusion criteria were kidney stone size ≥ 20 mm, or a history of PCNL failure. The imaging studies of the patients were evaluated by the operating surgeons and in cases where the possibility of success was deemed low by the operating surgeon (like scattered stones throughout the system), the case was scheduled for PCNL. The exclusion criteria were as follows: stones in locations which make them not accessible through pyelolithotomy incision (like stone in obstructed calyx or caliceal diverticula), need for urgent surgery, ascites, peritonitis, uncorrected coagulopathy, pyonephrosis, pregnancy, and severe ischemic heart disease.

LPL was performed as described below:

Following general anesthesia, the patient was placed in lateral decubitus position with minimal flexion. A 12-mm camera port was inserted through the umbilicus by an open access procedure. Under direct vision, three 5-mm ports were inserted in the midline about 10 cm lateral to the umbilicus, in the midclavicular line below the costal margins, and at the inferior abdomen lateral to the rectus muscles. Colon was reflected medially after incising the white line of Toldt. After identifying the ureter and pursuing its path to renal pelvis, the renal pedicle was exposed, followed by the removal of surrounding peripelvic fat up to the junction of the pelvis with the renal parenchyma. Using electrocautery or cold scissors, a U-shaped pyelotomy incision was made far from the ureteropelvic junction to avoid later stenosis of the ureteropelvic junction. In order to avoid excessive tearing of the pelvis, this incision was made on the renal pelvis as much as necessary in order to carefully extract the stone. The stone(s) were extracted from the ureteropelvic junction after being freed from the ureteropelvic junction using Maryland or Babcock graspers. As part of the procedure, a double pigtail ureteral stent was inserted into the renal pelvis, and the incision line was re-approximated using 4–0 Vicryl sutures in a running fashion. If continuous suturing was difficult, interrupted sutures were used.

Demographic and clinical data, operation characteristics, and postoperative data were extracted from the patients’ medical records. Preoperative ultrasonography (US) and computed tomography (CT) scan reports were reviewed to map the involved kidney, stone size, and location. Data regarding operative time, conversion to open surgery, duration of hospital stay, and postoperative outcomes such as bleeding, renal function loss, and fever were extracted from the records. Renal function was assessed based on plasma creatinine level measured before the procedure and on the first postoperative day. Blood loss was estimated based on the decrease in postoperative hemoglobin (Hb) level relative to its preoperative value. Complications were registered using Clavien-Dindo classification system during and after the procedure. Follow-up of patients included a clinic visit 2 weeks after the operation. All complications during hospitalization were extracted from hospital files.

The ethics of this study have been approved by the Ethical committee of the Urology and Nephrology Research Center with the following code: IR.SBMU.UNRC.REC.1400.003 and conducted in accordance with the tenets of the Declaration of Helsinki. All methods were carried out in accordance with relevant guidelines and regulations, and written informed consent was obtained from all patients or children’s parents/or their legal guardian(s) prior to the procedure.

### Statistical analysis

Continuous data are presented as the mean ± standard deviation (SD), and categorical data as numbers and percentages. Comparison of categorical data across different groups was performed using the Chi-square or Fisher’s exact test, as appropriate. In cases which the minimum calculated expected frequency was less than one or more than 20% of chi square table cells had expected frequencies less than 5, we used fisher exact test and in other cases Pearson chi square was used. The normality of continuous data was investigated by the Kolmogorov–Smirnov test. Comparison of normally distributed data across two groups was performed using an independent samples t-test and across more than two groups using one-way analysis of variance. Non-normally distributed data were compared using the Mann–Whitney U test for two independent groups and Kruskal–Wallis test for more than two independent groups. A P-value less than 0.05 was considered statistically significant.

## Results

### Participants

During 2003–2020, 451 patients (116 females and 335 males) with a mean ± SD (range) age of 46 ± 15 (1–92) years underwent LPL at Shahid Labbafinejad Hospital, Tehran, Iran.

During the first 6 years (2003–2008), only 15 LPL operations were performed. These operations were excluded from the analysis due to the initial learning curve and the limited number of yearly operations. From 2009 onward, yearly operations were more or equal to 17. We divided the time from 2009 to 2020 into three 4-year intervals: Group 1 (2009–2012), Group 2 (2013–2016), and Group 3 (2017–2020) and investigated the trend of LPL operations and their outcomes to investigate the influence of learning and maturation. There was no statistically significant difference between the time groups regarding the patients’ age, sex, stone size, and side of LPL (Table 1). However, the number of staghorn/multiple stones decreased from 49% in the initial 4 years to 20% in the last few years (p < 0.001). There were 12 children (age ≤ 15 years), 412 adults, and 12 geriatric patients (age ≥ 75 years). Abnormal anatomy was observed in 68 patients, including ureteropelvic junction obstruction (UPJO) in 26 patients (6.0%), horseshoe kidney in 23 patients (5.3%), ectopic/pelvic kidney in 17 patients (3.9%), and duplex or malrotated kidneys in 2 patients (0.4%). The most prevalent abnormality was UPJO and horseshoe kidney.

**Table 1:** Demographic and clinical characteristics of patients who underwent laparoscopic pyelolithotomy.

Variable	Whole series	Group1 (2009–2012)	Group2 (2013–2016)	Group3 (2017–2020)	P-value
Numbers	436	92	140	204	
Age, years; Mean ± SD	46.0 ± 15.4	47.1 ± 15.9	46.8 ± 15.9	45.5 ± 14.9	0.69
Gender, N; M/F	321/115	71/21	98/42	152/52	0.44
Side of LPL, N; L/R	230/206	54/38	74/66	102/102	0.38
Renal anatomy, N; Normal/Abnormal	368/68	86/6	123/17	159/45	**0.001**
Stone location, N; Single calyx/Single pelvis / Multiple or staghorn	32/271/133	4/43/45	15/78/47	13/150/41	** < 0.001**
Preoperative Creatinine, mg/dL; Median (IQR)	1.2 (1.1–1.5)	1.2 (1.0–1.5)	1.2 (1.0–1.4)	1.3 (1.1–1.5)	0.14
Preoperative Creatinine, N; Cr ≤ 1.5/1.51 < Cr < 3 / Cr ≥ 3	345/81/10	69/19/4	115/23/2	161/39/4	0.55
Preoperative Hb, mg/dL; Mean ± SD	13.9 ± 2.0	13.6 ± 2.0	13.9 ± 2.0	14.1 ± 2.0	0.24
Stone size, mm; Median (IQR)	30 (23–40)	30 (25–40)	30 (23–40)	30 (23–35)	0.09
Positive Hx of previous operation; N (%)	76 (17)	24 (26)	33 (24)	19 (9)	** < 0.001**

Significant values are in bold.

*N* number, *M* male, *F* female, *L* left, *R* right, *Hb* hemoglobin, *Hx* history, *SD* standard deviation.

### LPL operation characteristics and its outcomes

Thirteen LPL operations were converted to open surgery due to bleeding, severe adhesions, and the inability to remove a complete staghorn stone (Table S1). The outcomes of the patients who underwent LPL for the whole series as well as the groups according to the period of recruitment are presented in Table 2.

**Table 2:** Operative and postoperative data of patients who underwent laparoscopic pyelolithotomy.

Variable	Whole series	Group1 (2009–2012)	Group2 (2013–2016)	Group3 (2017–2020)	P-value
Number	436	92	140	204	
Hospitalization days; Mean ± SD	4.0 ± 2.1	4.5 ± 2.3	4.0 ± 2.2	3.6 ± 1.8	** < 0.001**
Operation duration, Minutes; Mean ± SD	158 ± 50	169 ± 51	155 ± 58	155 ± 42	**0.019**
Post-operative Creatinine, mg/dL; Mean ± SD	1.38 ± 0.67	1.54 ± 0.98	1.32 ± 0.54	1.23 ± 0.46	0.16
Post-operative Creatinine, N					
Cr ≤ 1.5 / 1.51 < Cr < 3 / Cr ≥ 3	331/95/10	61/27/4	109/29/2	161/39/4	0.14
Post-operative Hb, g/dL; Mean ± SD	12.6 ± 1.9	12.5 ± 1.7	12.5 ± 2.0	12.6 ± 1.9	0.77
Conversion to open surgery; numbers (%)	13 (3)	2 (2.2)	3 (2.1)	8 (3.9)	0.71
Fever; numbers (%)	125 (28.7)	33 (35.9)	47 (33.6)	45 (22.1)	**0.015**
Residual stone; numbers (%)	51 (11.7)	14 (15.2)	12 (8.6)	25 (12.3)	0.29
Clavien-Dindo grade of complication; numbers (%)					
No	249(57.1)	48 (52.2)	73 (52.1)	128 (62.7)	0.08*
Yes;					
1	92 (21.1)	24 (26.1)	39 (27.9)	29 (14.2)	
2	78 (17.9)	17 (18.5)	25 (17.9)	36 (17.6)	
3A	2 (0.5)	0 (0)	0 (0)	2 (1.0)	
3B	13 (3.0)	2 (2.2)	3 (2.1)	8 (3.9)	
4A	2 (0.5)	1 (1.1)	0 (0)	1 (0.5)	
Transfused PRBC; numbers (%)					
0	406 (93.1)	88 (95.7)	127 (90.7)	191 (93.6)	0.35
1	23 (5.3)	0	10 (7.1)	13 (6.4)	
2	3 (0.7)	1 (1.1)	2 (1.4)	0	
3	2 (0.5)	1 (1.1)	1 (0.7)	0	
4	2 (0.5)	2 (2.2)	0	0	
Significant leakage; numbers (%)	34 (7.8)	4 (4.3)	11 (7.9)	19 (9.3)	0.34

Significant values are in bold.

*N* number, *PRBC packed red blood cell.*

*Computed for No complication versus any grade of complication.

Fever was detected in 125 patients (28.7%) and 30 patients (7%) needed a blood transfusion. The number of packed red blood cell (PRBC) units transfused and the postoperative hemoglobin were not significantly different between time groups (P = 0.35 and P = 0.77, respectively). Table 2 reveals that patients who needed more than one unit of PRBC were operated on in the first 8 years of the study and in the last 4 years despite a significant increase in the total number of LPL operations, only 13 patients (6.4%) needed one unit PRBC and no patient needed more than one unit of PRBC.

The stone free rate (SFR) in the whole series was 88.3%. 51 patients (11.7%) had residual stones which were defined as any observable stone fragment with any size in postoperative imaging (CT scan or US). Interestingly SFR was not associated with time intervals of the study (P = 0.29). There was a significant decrease in the hospitalization duration over time (Table 2).

Twelve LPL operations were performed in children ≤ 15 years. Postoperative fever was observed in six children (50%) in comparison with 28% and 25% rates of postoperative fever for adults and geriatric patients in this study (P = 0.27). Nevertheless, no additional interventions were required for fever, and the patients were managed with antibiotics and hospitalized. The percentage of residual stones was 17% (two patients) in children versus 12% and 8% in adults and geriatric patients, respectively (P = 0.87). There was no difference in operation duration between children, adults, and geriatric patients (145.0 ± 55 vs. 154.0 ± 44 vs. 160.0 ± 58 min, respectively; P = 0.63). Likewise, there was no statistically significant difference in hospitalization duration among children, adults, and geriatric patients (P = 0.31). Minor complications (Clavian-Dindo grade 1–2) such as fever were higher in children but required no intervention and were cured with conservative treatment.

Another interesting finding of the current study is the outcome of LPL for kidneys with abnormal anatomies and solitary kidneys. There was no statistically significant difference in the size or location of stones between patients with normal and abnormal kidney anatomy. The frequencies (percentages) of multiple/staghorn stones in patients with normal versus abnormal anatomies were 117(31.8%) and 16(23.5%), respectively (P = 0.17). Likewise, the mean ± SD stone size in patients with normal versus abnormal anatomy was 31.6 ± 11.7 mm vs. 29.6 ± 11.7 mm respectively (P = 0.20). The findings of this study show no difference in operation duration, SFR, percent drop in postoperative creatinine or Hb, hospitalization, or complications between patients with normal versus abnormal kidney anatomy. A history of intervention for kidney stones was observed in 76 patients, including ESWL (N = 36), PCNL (N = 22), and open stone surgery (N = 18). As evident in Table 3, a history of stone intervention was not significantly associated with SFR, fever, operation duration, or complications. Likewise, the frequency (percentage) of urinary leakage in patients with no history of intervention, history of prior ESWL, history of prior PCNL, and history of prior open stone surgery was 27 (7%) vs. 2 (6%) vs. 2 (9%) vs. 3 (17%), respectively (P = 0.41). Also, the percent drops in 24-h postoperative Hb relative to preoperative values in patients with a negative history of stone intervention versus patients with ESWL history, PCNL history, and open stone surgery history were 9.3 ± 8.7 vs. 9.6 ± 8.0 vs. 7.1 ± 6.5 vs. 10.2 ± 14.2 respectively (P = 0.39). Interestingly, a history of PCNL was not associated with adverse outcomes in the investigated variables relative to patients with no history of intervention, whereas a history of open stone surgery was associated with a higher rate of adverse features in most investigated variables; however, this was not statistically significant in most instances. Nevertheless, patients with a history of open stone surgery had a longer hospitalization duration (P = 0.04).

**Table 3:** Characteristics and outcomes of patients who underwent laparoscopic pyelolithotomy.

	Stone free rate; number (%)		Fever; number (%)		Operation duration, minutes; Mean ± SD	Complication; number (%)		Hospitalization (days); Mean ± SD
	Yes	No	Yes	No		Yes	No	
Age categories								
Child	10 (83)	2 (17)	6 (50)	6 (50)	145 ± 55	7 (58)	5 (42)	4.0 ± 1.9
Adult	364 (88)	48 (12)	116 (28)	296 (72)	159 ± 50	176 (43)	236 (57)	4.0 ± 2.1
Geriatric	11 (92)	1 (8)	3 (25)	9 (75)	160 ± 58	4 (33)	8 (67)	4.1 ± 0.8
P-value	0.87		0.27		0.63	0.45		0.31
Stone location								
Single calyx/Single Pelvis	277 (91)	26 (9)	82 (27)	221 (73)	153 ± 49	124 (41)	179 (59)	3.8 ± 2.2
Multiple/Staghorn	108 (81)	25 (19)	43 (32)	90 (68)	171 ± 51	63 (47)	70 (53)	4.3 ± 2.0
P-value	0.002		0.26		< 0.001	0.21		0.05
Normal anatomy								
Yes	323 (88)	45 (12)	111 (30)	257 (70)	157 ± 51	158 (43)	210 (57)	4.1 ± 2.2
No	62 (91)	6 (9)	14 (21)	54 (79)	165 ± 45	29 (43)	39 (57)	3.5 ± 1.7
P-value	0.42		0.11		0.27	0.96		0.06
Solitary kidney								
Yes	7 (100)	0 (0)	1 (14)	6 (86)	178 ± 75	1 (14)	6 (86)	3.6 ± 1.0
No	378 (88)	51 (12)	124 (29)	305 (71)	158 ± 50	186 (43)	243 (57)	4.0 ± 2.2
P-Value	1.0		0.68		0.31	0.25		0.62
Pre-operative creatinine; mg/dl								
Cr ≤ 1.5	308 (89)	37 (11)	97 (28)	248 (72)	160 ± 51	146 (42)	199 (58)	3.9 ± 1.9
1.51 < Cr < 3	64 (85)	12 (15)	22 (27)	59 (73)	157 ± 48	33 (41)	48 (59)	3.9 ± 1.9
Cr ≥ 3	8 (80)	2 (20)	6 (60)	4 (40)	139 ± 47	8 (80)	2 (20)	7.6 ± 5.4
P-value	0.42		0.08		0.42	0.05		0.02
History of prior intervention for kidney stone;								
No	320 (89)	40 (11)	104 (29)	256 (71)	158 ± 49	154 (43)	206 (57)	3.9 ± 2.1
ESWL	30 (83)	6 (17)	12 (33)	24 (67)	160 ± 47	16 (44)	20 (56)	4.4 ± 2.1
PCNL	19 (86)	3 (14)	5 (23)	17 (77)	154 ± 36	8 (36)	14 (64)	3.3 ± 1.0
OSS	16 (89)	2 (11)	4 (22)	17 (77)	170 ± 81	9 (50)	9 (50)	5.1 ± 3.3
P-value	0.66		0.77		0.77	0.85		0.04

*Cr* creatinine, *ESWL* extracorporeal shock wave lithotripsy, *PCNL* percutaneous nephrolithotomy, *OSS* open stone surgery.

The last but not the least finding of our study was the outcomes of LPL for patients with a solitary kidney versus other patients. LPL was operated on 7 patients with a solitary kidney. Operations of solitary kidneys were performed more often in the initial years of the study (P = 0.04). All seven cases of LPL for solitary kidneys were performed on male patients (five on the left side and two on the right side). Stone location included two single pelvis stones and five cases of multiple/staghorn stones. Preoperative creatinine was ≥ 1.5 mg/dL in three cases (43%). Postoperative fever was observed in one of the seven patients which was managed by hospitalization and oral antibiotics without the need for any intervention. Transfusion was not necessary for any of these seven LPLs. The percent drop in postoperative creatinine and Hb was not different from that in patients without a solitary kidney (P = 0.27 and P = 0.96, respectively).

## Discussion

This was a large series of LPL operations from a single referral center over a long duration, reflecting the maturation process in this center and the selection of patients and their operative outcomes. Our findings reveal that with the passage of time, there has been a trend to exclude patients with staghorn/multiple stones or patients with high preoperative creatinine for LPL and to include a higher frequency of patients with abnormal anatomies. Operation duration and hospitalization time have decreased over time; however, the residual stone rate or complication frequency did not change over time. On the other hand, we observed a similar profile of LPL operation outcomes for geriatric patients compared to adults. The outcome of surgery for kidneys with abnormal anatomy (UPJO, horseshoe, ectopic, duplicated) was not different from kidneys with normal anatomy. We will discuss our prominent findings in the succeeding paragraphs on LPL and change in serum creatinine, LPL for staghorn/multiple stones, LPL on kidneys with abnormal anatomy, LPL on solitary kidneys, LPL for children or geriatric patients, LPL in patients with previous stone intervention, and LPL maturation through time respectively.

There are some concerns that the applied procedure for kidney stone removal might adversely affect kidney function. This concern applies mostly to open or laparoscopic stone surgeries with nephrotomy incisions or PCNL with multiple tracts^16^. We previously showed that the postoperative creatinine in PCNL shows a transient decrease and then returns to preoperative values within 48–72 h after the operation^17^. With respect to LPL, as there is no parenchymal incision, no deterioration of renal function is expected. In fact, our results revealed an average drop of 3.8% in the first postoperative day creatinine relative to its preoperative values. Interestingly, the percent drop in postoperative creatinine was higher in the more recent operations probably reflecting better case selection or improved skill. Few studies have examined the effect of laparoscopic or robotic pyelolithotomy on renal function. Swearegene et al. found no decrease in serum creatinine after robotic pyelolithotomy or nephrolithotomy^18^. Lee et al. also found no difference in serum creatinine between PCNL and LPL^19^. In a meta-analysis including 25 studies, Mao et al. reported an increase in glomerular filtration rate (GFR) of about 7.13 mL/min and a drop in postoperative creatinine of 0.03 mg/dL for LPL, which is very close to our findings^20^. Likewise, Schulster et al. reported an average 3 mL/min/1.73m2 increase in postoperative estimated GFR relative to its preoperative values in 16 patients who underwent robotic pyelolithotomy^21^. Another recent publication from our center averages the percent drop in serum creatinine 24 h after surgery about 7.7% compared with 3.8% presented above^22^. Compared to LPL operations on single stones, the results of the current study indicate that only operation duration and the rate of SFR were inferior in patients with staghorn or multiple stones. Other important features of the operation like complications, transfusion, and percent drop in postoperative Hb or creatinine were not different in patients with staghorn or multiple stones versus those with a single stone. The SFR in patients with staghorn or multiple stones was 81% which is higher than PCNL for staghorn stones and comparable to PCNL for non-staghorn stones, according to the global CROES study^23^. Few studies have evaluated the outcomes of LPL or robotic PL (RPL) for staghorn stones. The reported SFRs were 85%^13^, 88%^24^, and 85%^25^ in the series with limited numbers of patients. In two RCTs comparing LPL and PCNL, SFR was higher and bleeding lower, but the operation duration was longer^13,24^. We previously reported a randomized clinical trial on the outcomes of PCNL versus LPL for staghorn stones in which the stone free rate was 85% for LPL and 65% for PCNL (P < 0.05) showing the superiority of LPL for the clearance of staghorn stones^26^. Summarizing the above elaborations, LPL seems to play a feasible role in the treatment of staghorn stones.

There are limited reports on the outcome of LPL or RPL for patients with abnormal anatomy including ectopic, pelvic, malrotated, duplicated, or horseshoe kidneys. Most of these reports are case reports or case series including less than five patients. In this series, we have provided data on the outcome of LPL in 42 patients with abnormal anatomy. There was no difference in SFR, complications, transfusion, operation duration, hospitalization, and percent drop in postoperative Hb or creatinine between patients with abnormal anatomy and those with normal anatomy (Table 3). The literature also points to the feasibility of performing LPL or RPL in patients with UPJO^27^, horseshoe^28^, and pelvic/ectopic kidneys^29^. Furthermore, the above citations and the results of this study point to the safety and feasibility of performing LPL in selected patients with abnormal anatomy. The outcome of PCNL for the treatment of stones in kidneys with abnormal anatomy was previously reported by Gupta et al.^30^ They reported a success rate of 86% in horseshoe, ectopic, and malrotated kidneys which is marginally less than the success rate of LPL reported in the current study. Nevertheless, there are certain challenges on the performance of LPL in kidneys with abnormal anatomy. Sometimes it is difficult to distinguish the pelvis or pole of kidney which contains the stone for precisely planning the incision site. Using fluoroscopy or trying to touch the stone by a Shiba needle which is inserted transabdominally under ultrasonic vision and trying to touch the stone within kidney with the Shiba needle helps to verify the exact location of the stone within an ectopic or malrotated kidney. If after incising renal pelvis, no stone was observable, using a ureteroscope from the upper or lower abdominal trocars or advancement of the laparoscopic camera into the renal pelvicalyceal system and inspection of the lower, upper and middle calyces respectively will help to identify migrated stones.

We could not find any report on the outcome of LPL for solitary kidneys except for a few case reports. In fact, one case series reported the outcome of pyelolithotomy with pyeloplasty in patients with UPJO complicated by renal pelvis stone^31^. Our series included LPL operations on 7 patients with a solitary kidney. There were five solitary kidneys with multiple or staghorn stones and two solitary kidneys with a single stone. All patients with solitary kidneys were stone free after LPL and the rate of complications, transfusion, operation duration, and percent drop in postoperative creatinine or Hb in patients with solitary kidneys were not different from other patients. It seems that in carefully selected and well-informed patients, LPL is a feasible modality for the treatment of renal stones in solitary kidneys.

There are few reports on the performance of LPL in children or geriatric patients^32^. In the current study, we evaluated the influence of patients' age on the performance of LPL. There was no statistically significant relationship between SFR, transfusion, operation duration, hospitalization days, or percent drop in postoperative Hb or creatinine with the age of patients. Also, there was no statistically significant difference in the frequency of complications with patients’ age. The highest frequency of complications (mostly transient postoperative fever) was observed in the pediatric age group (58%) versus geriatric patients (33%). As stated in the results section, the reason for the observance of a high percentage of complications is that we considered any single measurement of T ≥ 38.3 °C as fever. Most of the complications were fever and managed with or without medications (Clavian Dindo grade ≤ 2). Nevertheless, the observance of a higher rate of low-grade complications in children warrants a more careful selection of children for LPL. Nevertheless, careful attention should be exerted to suction abdominal cavity fluids during the operation and to monitor postoperative electrolytes as children a more easily prone to electrolyte imbalance. In line with our findings, Hakan et al. reported that LPL is a safe and effective approach for children with large kidney stones in comparison with PCNL^33^.

Over 90% of patients with prior abdominal surgery develop adhesions, which can cause challenges in laparoscopic surgery or increase the risk of complications^34,35^. Few studies have addressed the feasibility of urological laparoscopic procedures such as pyeloplasty or nephrectomy in patients with a prior history of renal interventions. Wang et al. found laparoscopic nephrectomy was feasible in such patients^36^. Likewise, Radfar et al. reported no difference in SFR, operating time, hospitalization, and complications for LPL between patients with previous kidney interventions and a control group^37^. Our study included 76 patients who had a history of prior kidney intervention for stone disease which is one of the largest in the literature. Interestingly, LPL had no adverse effects on complications or operation duration in patients with prior PCNL.

This report evaluates the performance and maturation of LPL over a long period of time. The results show that with the passage of time, there has been a decrease in hospitalization duration, operation duration, and post-operative creatinine suggesting the maturation of surgeries. Nevertheless, there have been no changes in the more important aspects of the operation, such as leakage, complications, and SFR. This result points to the fact that the performance of LPL can be safe even with moderate expertise.

In a recent meta-analysis comparing LPL, RPL, and PCNL, the mean operative time was significantly shorter in favor of PCNL. Paradoxically, the mean estimated blood loss was higher and SFR was lower in the PCNL. In addition, the study revealed no significant difference among the three surgical approaches in terms of length of stay, complications, failure, and conversion rate^38^.

The limitations of this study are worth mentioning. This was a single-center retrospective study of prospectively gathered data. There are biases relevant to retrospective studies in our study, most notably the selection and recall bias. Further prospective studies with comparator groups and long-term follow-up are recommended to verify the results of the current study. Another limitation is the absence of a comparison group. We compared the results of LPL in this report with our previous reports on PCNL or publications of other researchers. Nevertheless, this study provides data on a large scale and range of patients over a long period of time and sheds light on some specific properties of the LPL operation that have not been investigated before.

## Conclusion

This study included a large number of cases with extensive diversity in age, stone burden, and renal anatomy. Performing LPL is feasible in carefully selected geriatric patients with similar SFR and complication rates in adult patients. The outcomes of LPL for kidneys with staghorn or multiple stones are satisfactory. LPL can also be applied for kidneys with abnormal anatomy or for solitary kidneys. The authors conclude that LPL can be considered in any age group and in patients with kidney abnormalities as an alternative surgical approach to PCNL or RIRS, given that relevant expertise is available.

### Supplementary Information

Supplementary Table S1.

## Data Availability

The datasets used and/or analyzed during the current study are available from the corresponding author on reasonable request.
