# Supplementary material for: The experience of a tertiary referral center with laparoscopic pyelolithotomy for large renal stones during 18 years
Source: Sci Rep. 2023 Dec 28;13:23102. doi: 10.1038/s41598-023-50331-w (PMC10754888; doi:10.1038/s41598-023-50331-w)
Supplement: Supplementary file 1 — Supplementary Table S1. [file 41598_2023_50331_MOESM1_ESM.docx]

**Table S1: Details of patients converted from laparoscopy to open surgery**

| Number | Age | Sex | Stone location | Stone size  (mm) | Stone side | Previous operation | Operation duration(min) | Preop Hb | Postop Hb | Hospitalization  (day) | Details |
| --- | --- | --- | --- | --- | --- | --- | --- | --- | --- | --- | --- |
| 1 | 15 | Male | Single pelvis | 20 | Left | No | 180 | 13 | 12.3 | 4 | Inaccessible stone |
| 2 | 35 | Male | Multiple or staghorn | 80 | Right | No | 300 | 14.5 | 11.5 | 8 | Excessive bleeding |
| 3 | 58 | Male | Multiple or staghorn | 38 | Left | No | 250 | 14.4 | 12.4 | 3 | Excessive bleeding |
| 4 | 57 | Male | Single pelvis | 34 | Left | No | 290 | 14.2 | 11.6 | 4 | Inaccessible stone due to kidney malformation |
| 5 | 36 | Female | Multiple or staghorn | 65 | Left | No | 150 | 13.2 | 9.7 | 4 | UPJ avulsion due to adhesion |
| 6 | 36 | Male | Single pelvis | 45 | Left | No | 125 | 16.5 | 14 | 3 | failure to progress due to adhesions |
| 7 | 31 | Female | Single pelvis | 10 | Left | Yes | 130 | 11.3 | 9.5 | 5 | excessive abdominal adhesions from the previous operation |
| 8 | 46 | Male | Single pelvis | 28 | Right | No | 130 | 14.9 | 11.8 | 3 | coverage of anterior pelvis surface by renal vessels |
| 9 | 33 | Female | Single pelvis | 30 | Left | No | 165 | 12.9 | 9 | 7 | coverage of anterior pelvis surface by renal vessels |
| 10 | 52 | Male | Single pelvis | 16 | Right | No | 210 | 14.3 | 12.4 | 3 | failure to place ureteral catheter through laparoscopy |
| 11 | 46 | Male | Single pelvis | 23 | Left | No | 130 | 11.4 | 10.8 | 4 | failure to progress due to adhesions |
| 12 | 40 | Male | Multiple or staghorn | 45 | Right | No | 150 | 11.7 | 9.1 | 3 | active drainage after surgery, bleeding from laparoscopic port vessels |
| 13 | 41 | Male | Single calyceal | 18 | Left | No | 210 | 15.9 | 10.2 | 5 | Inaccessible stone |
